# Supplementary material for: Immuno-metabolic stress responses control longevity from mitochondrial translation inhibition in C. elegans
Source: Nat Commun. 2025 Jul 2;16:6083. doi: 10.1038/s41467-025-61433-6 (PMC12222763; doi:10.1038/s41467-025-61433-6)
Supplement: Supplementary file 1 — Supplementary Information [file 41467_2025_61433_MOESM1_ESM.pdf]

Supplementary Information

Immuno-metabolic stress responses control longevity from mitochondrial translation inhibition in *C. elegans*

Iman Man Hu[1,2], Marte Molenaars[6], Yorrick R.J. Jaspers[1,2], Bauke V. Schomakers[1,2,4], Michel van Weeghel[1,2,4], Amber Bakker[1], Melanie Modder[1], Joseph P. Dewulf[5], Guido T. Bommer[5], Arwen W. Gao[1,2], Georges E. Janssens[1,2], Riekelt H. Houtkooper[1,2,3, #]

[1]*Laboratory Genetic Metabolic Diseases, Amsterdam UMC Location University of Amsterdam, Meibergdreef 9, Amsterdam, The Netherlands,*

[2]*Amsterdam Gastroenterology Endocrinology and Metabolism Institute, Amsterdam, The Netherlands,*

[3]*Amsterdam Cardiovascular Sciences Institute, Amsterdam, The Netherlands,*

[4]*Core Facility Metabolomics, Amsterdam UMC Location University of Amsterdam, Meibergdreef 9, Amsterdam, The Netherlands,*

[5]*Department of Biochemistry, de Duve Institute, UCLouvain, Brussels, Belgium,*

[6]*Department of Pathology, New York University Grossman School of Medicine, New York, NY 10016, USA.*

[#] *Correspondence: r.h.houtkooper@amsterdamumc.nl*

Supplementary Figures

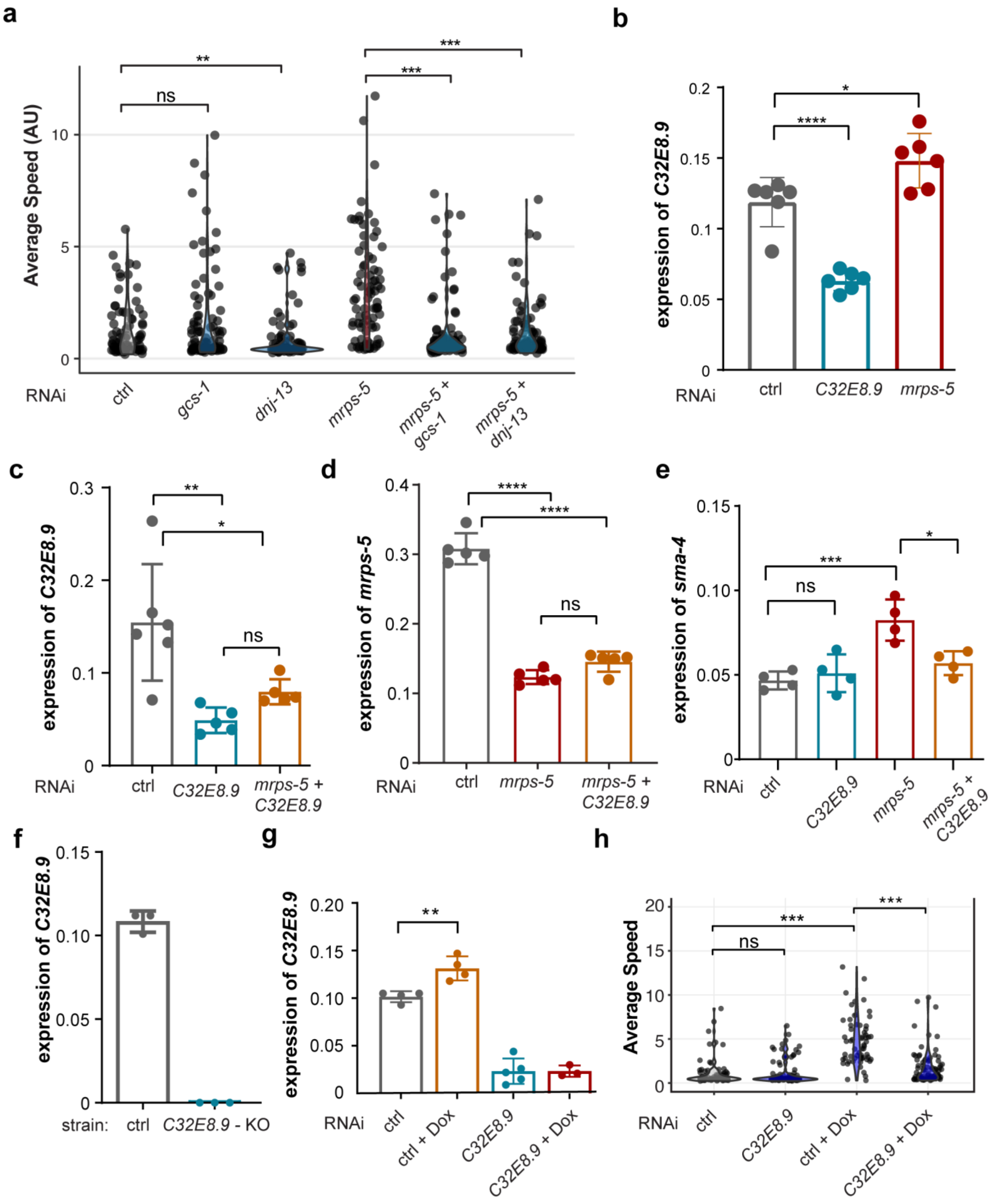

Fig S1 | Healthspan of candidates and expression of *C32E8.9* in different conditionsa Violin plot representing the average moving speed of GMC101 worms under control (HT115), *gcs-1* RNAi, *dnj-13* RNAi, *mrps-5* RNAi, *mrps-5* + *gcs-1* RNAi, *mrps-5* + *dnj-13* RNAi bacteria at day 4 of adulthood. ** represents wilcox.test p-value < 0.01, *** p-value < 0.001, "ns" represents not significant**.** AU: Arbitrary Units. **b-c** Relative expression of *C32E8.9* measured by qPCR in N2 worms treated with various RNAi bacteria. **d** Relative expression of *mrps-5* measured by qPCR in N2 worms treated with various RNAi bacteria. **e** Relative expression of *sma-4* measured by qPCR in N2 worms treated with various RNAi bacteria. For panels b-e, statistical analysis was performed using the Analysis of Variance (ANOVA) test. Significance levels are denoted as follows: *** represents p-value < 0.001, ** represents p-value < 0.01, and * represents p-value < 0.05. **f** Relative expression of *C32E8.9* measured by qPCR in control worms (N2 background strain) and *C32E8.9* knockout (KO) worms. **g** Relative expression of *C32E8.9* measured by qPCR in N2 worms treated with control HT115 bacteria, *C32E8.9* RNAi bacteria, control + Dox (doxycycline), or Dox + *C32E8.9* RNAi. Statistical analysis was performed using the Analysis of Variance (ANOVA) test. ** represents p-value < 0.01. **h** Violin plot representing the average moving speed of GMC101 worms under control (HT115), *C32E8*.9 RNAi, control + Dox and *C32E8.9* RNAi + Dox treatments at day 4. *** represents wilcox.test p-value < 0.001.

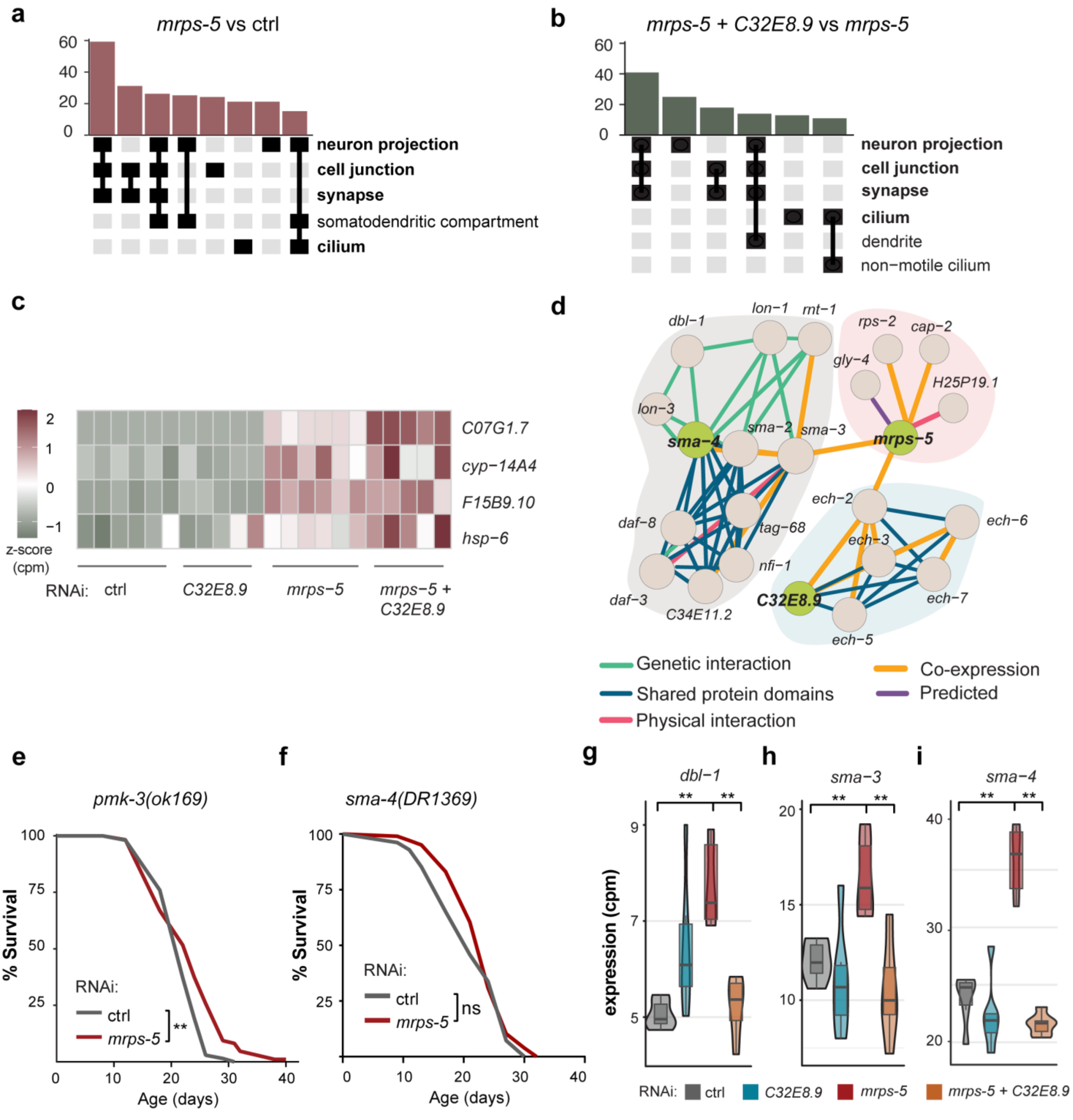

Fig S2 | *C32E8.9* reversed the immune response activated by mitochondrial translation inhibition

a Upset plot of top enriched cellular component genesets in significantly up-regulated genes (adjust p-value <0.05, $\log_2 FC > 0.5$) in *mrps-5* RNAi vs control. The size of bar represents the number of genes overlapping among gene sets. **b** Upset plot of top enriched cellular component genesets in significantly down-regulated genes (adjust p-value < 0.05, $\log_2 FC < -0.5$) in *mrps-5* + *C32E8.9* double RNAi vs *mrps-5* RNAi. The size of bar represents the number of genes overlapping among gene sets. Terms appearing in both panel (**a**) and panel (**b**) are highlighted in bold. **c** Heatmap of mRNA expression of genes in UPR^{mt} in four RNAi knockdown conditions. Fill color represents the Z-score transformed counts per million (CPM) gene expression for each gene across all samples. For visualization purposes, values exceeding 2 were capped at 2, while values

below -2 were capped at -2. **d** Network generated from GeneMANIA showing that no direct associations were reported between *sma-4*, *C32E8.9* and *mrps-5*. Green nodes represent genes of interest. **e-f** Lifespan measurements in *pmk-3(ok169)* and *sma-4(DR1369)* mutants. Worms were fed with control (solid gray line) or *mrps-5* RNAi (solid red line). For panels (e) and (f), p-values were calculated using the log-rank test to compare each condition with the corresponding control. In panel **e**, 222 – 248 worms were analyzed per condition across two independent experimental replicates, as detailed in Supplementary Table S1. In panel **f**, 165 – 168 worms were analyzed per condition across two independent experimental replicates, as detailed in Supplementary Table S1. Statistical significance is indicated as follows: ** represents p-value < 0.01, "ns" represents not significant. **g-i** mRNA expression of genes in TGF-β pathway. Fill color represents the RNAi condition. Statistical analysis was performed using the Analysis of Variance (ANOVA) test. *** represents p-value <0.001, **** represents p-value < 0.0001, "ns" represents not significant.

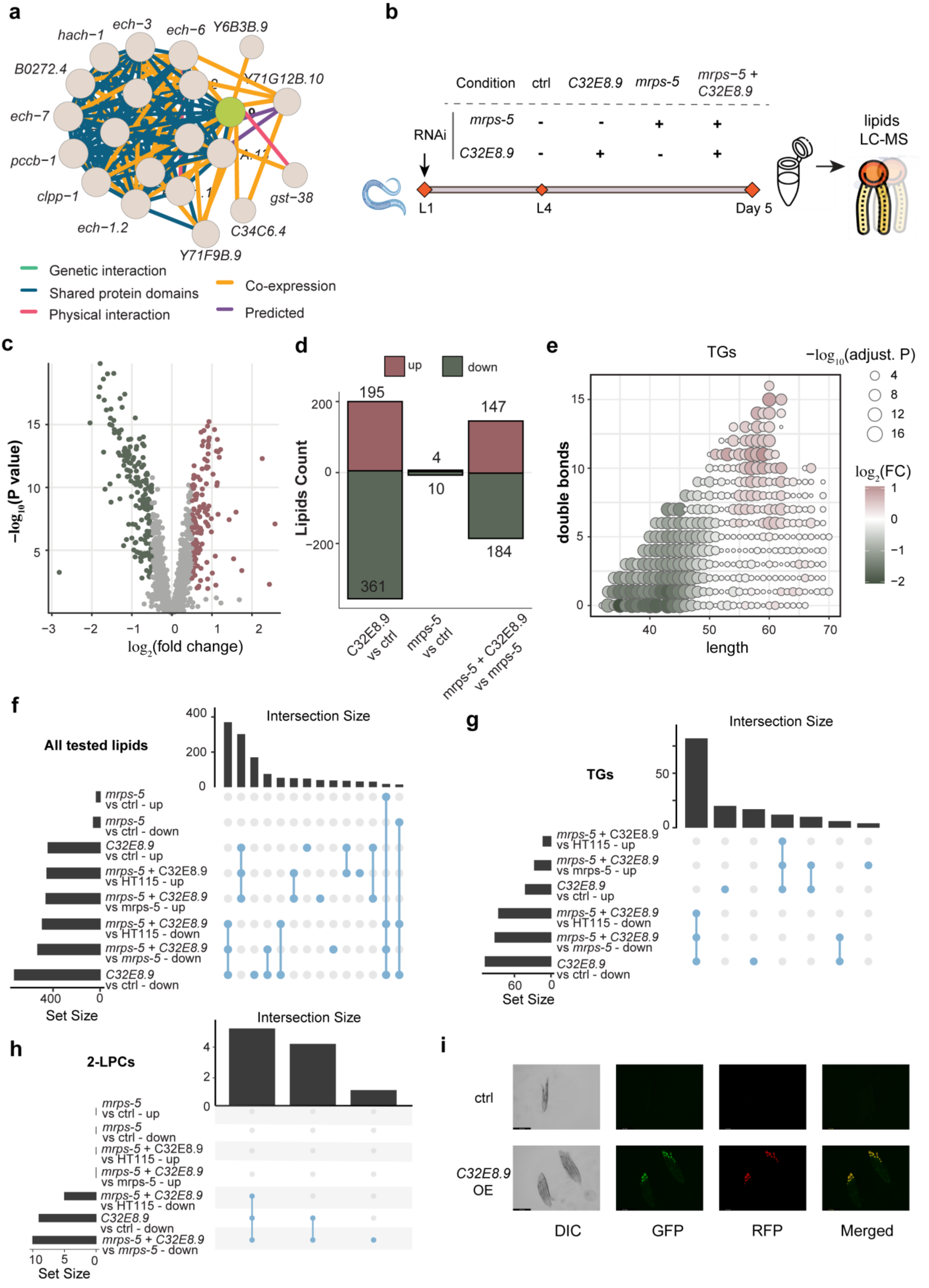

Fig S3 | *C32E8.9* mediates lipidomic changes under mitochondrial translation inhibition.

a GeneMANIA interaction network of *C32E8.9*. **b** Experimental design of lipidomics. **c** Volcano plot of lipids in *mrps-5* + *C32E8.9* double RNAi vs *mrps-5* RNAi (adjusted p-value < 0.05, absolute $\log_2$FC > 0.5). **d** Bar plot of the number of significantly altered lipids (absolute $\log_2$FC > 1) in different comparisons. **e** Dot plot of all DGs in *mrps-5* + *C32E8.9* double RNAi vs *mrps-5* RNAi comparison. The X-axis represents the length of DGs. The Y-axis represents the number of double bonds of DGs. **f - h** UpSet plots illustrating the overlap of tested lipids (**f**), triglycerides (TGs) (**g**), and 2-lysophosphatidylcholines (2-LPCs) (**h**) across different comparisons. Bar heights represent the number of overlapping lipids, TGs, and 2-LPCs respectively. **i** Representative fluorescence images of control worms (N2 background strain) and the *C32E8.9* overexpression (OE) strain at day 1. The genotype of *C32E8.9*-OE strain is *Pdpy-30*-*C32E8.9* cDNA-3xGGSG-GFP-unc-54 3'UTR.

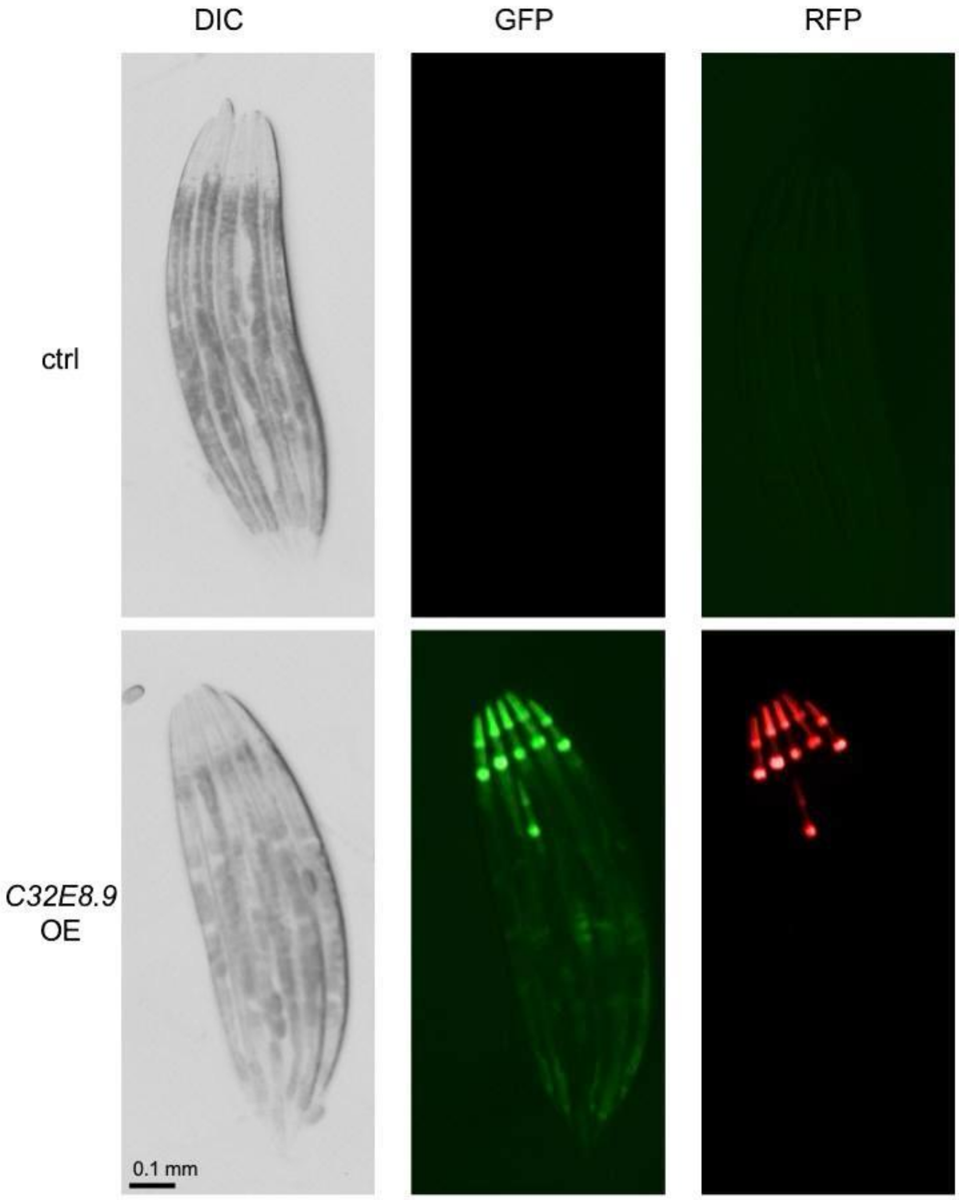

Fig S4 | Representative high-magnification images.

High-magnification images of control worms (N2 background strain) and the *C32E8.9* overexpression (OE) strain at day 1. The genotype of *C32E8.9*-OE strain is *Pdpy-30-C32E8.9* cDNA-3xGGSG-GFP-unc-54 3'UTR.

Table S1: Details for PA01 slow-killing assays

C. elegans strain	Treatment	Median lifespan (hours)	Maximum lifespan (hours)	Number of tested animals	number of replicates	p – value versus control group
**Fig 5b**						
Wild Type (N2)	HT115	116	166	86	Replicate 1*	
	20% *mrps-5* + 80% HT115	142	180	85		+***
	80% *C32E8.9* + 20% HT115	116	166	87		+ ns
	20% *mrps-5* + 80% *C32E8.9*	116	166	87		# *
Wild Type (N2)	HT115	124	148	109	Replicate 2	
	20% *mrps-5* + 80% HT115	124	172	108		+ *
	80% *C32E8.9* + 20% HT115	124	172	107		+ ns
	20% *mrps-5* + 80% *C32E8.9*	124	148	108		# *
**Fig 5c**						
Control	HT115	95	174	85	Replicate 1*	
	20% *mrps-5* + 80% HT115	126	185	85		+ *
C32E8.9 KO	HT115	95	174	84		
	20% *mrps-5* + 80% HT115	95	174	84		+ ns
Control	HT115	120	145	84	Replicate 2	
	20% *mrps-5* + 80% HT115	120	170	85		+ *
C32E8.9 KO	HT115	95	170	87		
	20% *mrps-5* + 80% HT115	120	170	85		+ ns
**Fig 5d**						
Wild Type (N2)	HT115	116	166	86	Replicate 1*	
	20% *mrps-5* + 80% HT115	142	180	85		+ **
	80% *sma-4* + 20% HT115	116	166	90		+ ns
	20% *mrps-5* + 80% *sma-4*	116	166	89		# 0.08
Wild Type (N2)	HT115	124	148	109	Replicate 2	
	20% *mrps-5* + 80% HT115	124	172	108		+ *
	80% *sma-4* + 20% HT115	96	148	108		+ ns
	20% *mrps-5* + 80% *sma-4*	124	148	106		# *
**Fig7 b**						
Control	OP50	95	152	84	Replicate 1*	
C32E8.9 OE	OP50	126	174	84		+ **
Control	OP50	95	145	86	Replicate 2	
C32E8.9 OE	OP50	120	170	84		+ **

*Experiment represented in figure. + p-value from Log-rank test compared to control. # p-value from Log-rank test compared to *mrps-5* RNAi. **** represents p-value < 0.0001, *** represents p-value < 0.001, **** represents p-value < 0.0001, "ns" represents not significant.
