# Supplementary material for: Immuno-metabolic stress responses control longevity from mitochondrial translation inhibition in C. elegans
Source: Nat Commun. 2025 Jul 2;16:6083. doi: 10.1038/s41467-025-61433-6 (PMC12222763; doi:10.1038/s41467-025-61433-6)
Supplement: Supplementary file 2 — Description of Additional Supplementary Files [file 41467_2025_61433_MOESM2_ESM.pdf]

Description of Additional Supplementary Files

Supplementary Data 1: Details of lifespan.
