# Supplementary material for: Immuno-metabolic stress responses control longevity from mitochondrial translation inhibition in C. elegans
Source: Nat Commun. 2025 Jul 2;16:6083. doi: 10.1038/s41467-025-61433-6 (PMC12222763; doi:10.1038/s41467-025-61433-6)
Supplement: Supplementary file 4 — Reporting Summary [file 41467_2025_61433_MOESM4_ESM.pdf]

nature portfolio

Corresponding author(s): Riekelt H. Houtkooper

Last updated by author(s): April 16, 2025

Reporting Summary

Nature Portfolio wishes to improve the reproducibility of the work that we publish. This form provides structure for consistency and transparency in reporting. For further information on Nature Portfolio policies, see our Editorial Policies and the Editorial Policy Checklist.

Statistics

For all statistical analyses, confirm that the following items are present in the figure legend, table legend, main text, or Methods section.

n/a	Confirmed	
☐	☒	The exact sample size (*n*) for each experimental group/condition, given as a discrete number and unit of measurement
☒	☐	A statement on whether measurements were taken from distinct samples or whether the same sample was measured repeatedly
☐	☒	The statistical test(s) used AND whether they are one- or two-sided<br>*Only common tests should be described solely by name; describe more complex techniques in the Methods section.*
☒	☐	A description of all covariates tested
☐	☒	A description of any assumptions or corrections, such as tests of normality and adjustment for multiple comparisons
☐	☒	A full description of the statistical parameters including central tendency (e.g. means) or other basic estimates (e.g. regression coefficient) AND variation (e.g. standard deviation) or associated estimates of uncertainty (e.g. confidence intervals)
☐	☒	For null hypothesis testing, the test statistic (e.g. *F*, *t*, *r*) with confidence intervals, effect sizes, degrees of freedom and *P* value noted<br>*Give P values as exact values whenever suitable.*
☒	☐	For Bayesian analysis, information on the choice of priors and Markov chain Monte Carlo settings
☒	☐	For hierarchical and complex designs, identification of the appropriate level for tests and full reporting of outcomes
☐	☒	Estimates of effect sizes (e.g. Cohen's *d*, Pearson's *r*), indicating how they were calculated

Our web collection on statistics for biologists contains articles on many of the points above.

Software and code

Policy information about availability of computer code

Data collection

Lipidomics data were generated by UPLC-mass spec.
RNA-seq data were generated by using KAPA mRNA HyperPrep Kit and Illumina NovaSeq

Data analysis

FastQC
fastp version 0.23.2
STAR2 version 2.5.4
R version 4.1.1
DESeq2 version1.32.0
edgeR version 3.36.0
Clusterprofiler version 4.0.5
org.Ce.eg.db version 3.13.0
ggplot2 version 3.4.2

All code related to RNAseq and lipidomics data analysis has been deposited on GitHub. Readers can access it via the following link: https://github.com/ManHUU/C32E8.9

For manuscripts utilizing custom algorithms or software that are central to the research but not yet described in published literature, software must be made available to editors and reviewers. We strongly encourage code deposition in a community repository (e.g. GitHub). See the Nature Portfolio guidelines for submitting code & software for further information.

Data

Policy information about availability of data

All manuscripts must include a data availability statement. This statement should provide the following information, where applicable:

- Accession codes, unique identifiers, or web links for publicly available datasets
- A description of any restrictions on data availability
- For clinical datasets or third party data, please ensure that the statement adheres to our policy

.RNA-seq data has been deposited in GEO. Reviewers can access it with the accession number "GSE248642" (https://www.ncbi.xyz/geo/query/acc.cgi?acc=GSE248642). The lipidomics data generated in this study are provided in the Source Data file accompanying this paper. All other data supporting the findings of this study are available from the corresponding author upon reasonable request.

Research involving human participants, their data, or biological material

Policy information about studies with human participants or human data. See also policy information about sex, gender (identity/presentation), and sexual orientation and race, ethnicity and racism.

Reporting on sex and gender	N/A
Reporting on race, ethnicity, or other socially relevant groupings	N/A
Population characteristics	N/A
Recruitment	N/A
Ethics oversight	N/A

Note that full information on the approval of the study protocol must also be provided in the manuscript.

Field-specific reporting

Please select the one below that is the best fit for your research. If you are not sure, read the appropriate sections before making your selection.

☒ Life sciences ☐ Behavioural & social sciences ☐ Ecological, evolutionary & environmental sciences

For a reference copy of the document with all sections, see nature.com/documents/nr-reporting-summary-flat.pdf

Life sciences study design

All studies must disclose on these points even when the disclosure is negative.

Sample size	For RNA-sequencing, 5-6 samples per condition, around 1000 worms per sample.<br>For lipidomics, 5-6 samples per condition, around 2000 worms per sample.
Data exclusions	Data was not excluded.
Replication	All experiments were replicated at least twice, except for large scale omics data.
Randomization	RNA-sequencing and lipidomics were randomized.
Blinding	Blinding was not possible in this study because the group allocation, the data collection and data analysis were for most of the measurements performed by the same investigator. Key lifespan experiments of C32E8.9 RNAi were confirmed by multiple investigators. Lipidomics were analyzed by other investigators of the research team who were blinded for group allocations.

Reporting for specific materials, systems and methods

We require information from authors about some types of materials, experimental systems and methods used in many studies. Here, indicate whether each material, system or method listed is relevant to your study. If you are not sure if a list item applies to your research, read the appropriate section before selecting a response.

Materials & experimental systems

n/a	Involved in the study	
☒	☐	Antibodies
☒	☐	Eukaryotic cell lines
☒	☐	Palaeontology and archaeology
☐	☒	Animals and other organisms
☒	☐	Clinical data
☒	☐	Dual use research of concern
☒	☐	Plants

Methods

n/a	Involved in the study	
☒	☐	ChIP-seq
☒	☐	Flow cytometry
☒	☐	MRI-based neuroimaging

Animals and other research organisms

Policy information about studies involving animals; ARRIVE guidelines recommended for reporting animal research, and Sex and Gender in Research

Laboratory animals	C. elegans.<br>Strains used in this study include: Bristol N2, CB1370 [daf-2(e1370)], DA465 [eat-2(ad465)], KU25 [pmk-1(km25)], BS3383 [pmk-3(ok169)], FK171 [mek-1(ks54)/sek-1(qd127)], SJ4100 [zcIs13[hsp-6p::gfp + lin-15(+)]], GMC101 [dvIs100 (unc-54p::A-beta-1-42::unc-54 3'-UTR + mtl-2p::GFP)].
Wild animals	No wild animals were used.
Reporting on sex	N/A
Field-collected samples	N/A
Ethics oversight	N/A

Note that full information on the approval of the study protocol must also be provided in the manuscript.

Plants

Seed stocks	N/A
Novel plant genotypes	N/A
Authentication	N/A
